# Effect of process standards on survival of patients with head and neck cancer in the south and west of England

**DOI:** 10.1038/sj.bjc.6602118

**Published:** 2004-10-05

**Authors:** M Birchall, D Bailey, P King

**Affiliations:** 1 University of Liverpool, University Hospital Aintree, Liverpool L9 7AL, UK; 2 South West Cancer Intelligence Service, Winchester SO22 5DH, UK

**Keywords:** head and neck cancer, standards, survival, waiting times

## Abstract

The aim of the study was to compare standards for the process of care and 2-year survival between two cohorts of patients with head and neck cancer in the south and west of England. A total of 566 and 727 patients presented in 1996–97 and 1999–2000, respectively. The median number of cases treated per surgeon was 4 (1997, range 1–26) and 4 (2000, 1–23) and per radiotherapist was 10 (1–51) and 19 (1–70). For all ‘nontemporal’ standards, the overall standard increased, without reaching minimum high targets, while most ‘waiting times’ increased. Overall 2-year survival was 64.1% in 1997 and 65.1% in 2000. There was no difference in survival between networks (range 56–68, 1997, log-rank test 4.1, *P*=0.4; 62–69, 2000, log-rank test 1.26, *P*=0.69). Patients assessed by a multidisciplinary clinic exhibited improved survival (1997: *P*=0.1; 2000: hazard ratio 0.7, *P*=0.02), as did those with a pretreatment chest X-ray (hazard ratio 0.7, *P*=0.03). Despite an increased incidence, standards for the process of care for patients with head and neck cancer improved between 1996 and 2000, while waiting times increased and 2-year survival rates remained unaltered. Two out of five networks demonstrated centralisation of services between audits. Being seen in a multidisciplinary clinic correlated strongly with patient survival.

In 1998, we released the first report of the South and West Audit of Head and Neck Cancer, SWAHN I (Birchall *et al*, 2000). Data collection for this had begun in 1996 and clinicians and hospitals barely had time to respond to the changes recommended by the Calman–Hine report (Expert Advisory Group on Cancer, 1995). Those changes were not clearly stated, and there was widespread scepticism that clinicians had been doing things wrongly in the first place and there was no explicit funding for change. This audit, based on standards developed by a formal consensus method (Birchall, 1998; Wilson, 2003), benchmarked how UK head and neck services were performing in the period leading up to the ‘reforms’. Since then, central funding has been released with the intention of improving standards in cancer care (Department of Health, 2002), although how much of this has reached head and neck services on the ground is unclear.

A repeat of the audit in 1999–2000, SWAHN II (Birchall and Bailey, 2001), presented the opportunity to examine whether standards in head and neck cancer care in the UK had improved post-Calman–Hine and whether this was accompanied by improved survival.

## MATERIALS AND METHODS

The same population (6.5 millions) was examined by both audits, with the exception of one hospital, which had only diagnosed three cases in the first study. ‘Cases’ were all those patients diagnosed with a primary head and neck cancer between 1st December 1996 and 30th November 1997 (SWAHN I) and 1st September 1999 to 31st August 2000 (SWAHN II). Skin, lip, thyroid cancer and lymphomas were excluded.

New cases were identified by clinicians within each Trust and details recorded on a paper proforma that included demographic details and fields corresponding to each of the consensus standards for the process of care (Birchall, 1998). Internal audit and peer-review methods assured accuracy and validity of information received. Computerised validation checks identified erroneous data entries. Comparison with the Cancer Register and with pathology records for all cancer cases, routinely forwarded monthly by each Trust, was performed. Where further cases found, reminders were sent to the relevant clinician. A final quality check was performed by arbitrary sampling of case notes. In 2003, lists of all cases entered into the audit were sent to Trusts. Information was requested on whether the patient was alive or dead, date of loco-regional recurrence and death and recorded cause of death. Deaths were then crosschecked with the cancer registry.

Kaplan–Meier survival analysis was carried out (SPSS, SPSS Corp., USA). Univariate analysis for significant differences in distribution between groups used log-rank tests. Multivariate analysis of survival against case-mix and standards used Cox proportional hazards.

## RESULTS

### Distribution of cases

The number of cases for which complete data were obtained increased from 566 in 1997 to 727 in 2000, that is, an increase of 29%. The number of cases predicted from registry figures for the corresponding years was 650 and 768 respectively (an increase of 18%), giving predicted capture rates of 87 and 95% respectively (an increase of 8% between audits). Male : female distribution remained at 2 : 1, but there was a slight trend towards younger age-groups (45–64 group increased from 34 to 38%, 65–74 group decreased from 32 to 26%). There was a nonsignificant increase in oral cancer (30–33%) and a decrease in laryngeal cancer (32–26%), but no change in the proportion of cases with each UICC stage.

### Treatment

Proportions receiving surgery (50 and 47%), radiotherapy (69 and 68%) and chemotherapy (7.4 and 8.5%; Table 1

**Table 1:** Chemotherapy use in SWAHN I and SWAHN II, showing intent and method of treatment

**Treatment intent**	**Total number**	**Number neoadjuvant**	**Number concurrent**	**Number single modality**
SWAHN I				
Palliative	10	3	4	3
Curative	29	12	14	3
Not stated	3	1	2	1
Total	42	16	20	7
				
SWAHN II				
Palliative	16	8	3	5
Curative	38	7	25	6
Not stated	8	3	3	2
Total	62	18	31	13

) were similar. There was a nonsignificant increase in use of radiotherapy as sole treatment for laryngeal and pharyngeal cancers (80 and 50% in SWAHN I compared to 95 and 70% in SWAHN II). The use of partial (mainly endoscopic) surgery for laryngeal tumours increased from 20% of operations to 25%. The median number of cases treated per surgeon in 1997 was 4 (range 1–26) and this was unchanged in 2000 (4, 1–23). However, the number per consultant radiotherapist increased from 10 (1–51) to 19 (1–70) between audits. The degree of ‘centralisation’ of treatment between 1997 and 2000 varied considerably between networks. For central south coast, the number of treating hospitals decreased from six to two and for Dorset from two to one, whereas the other three networks showed no obvious change in referral patterns (Figure 1

**Figure 1:**
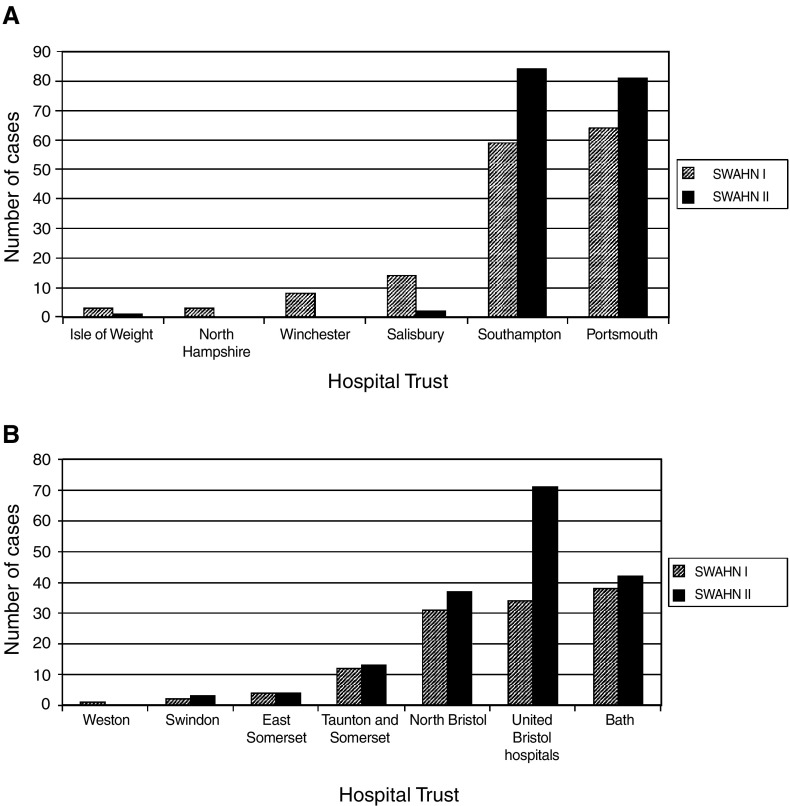
Number of head and neck cancer patients diagnosed and treated by hospital Trust in (**A**) central south coast and (**B**) Avon Somerset and Wiltshire cancer networks. (**A**) shows evidence of centralisation, while (**B**) does not. Other networks showed similar results to (**B**).

).

### Standards

For all ‘nontemporal’ standards, the overall standard increased, although without reaching the established ‘minimum high’ targets (defined as ‘the lowest level compatible with something that a body of experts would regard as high quality care’; Table 2

**Table 2:** Comparison between nontemporal standards achieved and target standards in SWAHN I and SWAHN II

**Standard**	**SWAHN I**		**SWAHN II**		**Target standard (%)**
	***n***	**%**	***n***	**%**	
Patients seen at a combined clinic	566	46	727	74	95
					
Having an X-ray	566	52	727	68	100
					
Advanced T3/T4 larynx and oral tumours having a pretreatment MRI/CT scan	126	56	156	78	90
					
TNM staging	566	83	727	87	100

MRI=magnetic resonance imaging; CT=computerised tomography; TNM=tumour, node, metastasis.

). Most marked was the increase in the proportion of patients assessed at multidisciplinary clinics (46–74%, standard 95%). These were defined as clinics where the patient was assessed by at least one consultant oncologist and radiotherapist and one head and neck surgeon. Pretreatment chest X-rays also increased from 52 to 68% (standard 100%), while the proportion of advanced (T3/T4) stage tumours receiving MRI or CT scan grew from 56 to 78% (target 90%). In 83% of cases in 1997 and 87% in 2000 (target 100%), tumour, node, metastasis (TNM) staging was achieved.

### Times between activities

A consensus pathway for the process of care and standards for the intervals along this pathway had previously been determined (Birchall, 1998). For a few parts of the pathway, ‘waiting’ times remained constant, but most increased between 1997 and 2000 (Table 3

**Table 3:** Comparison between temporal standards achieved and target standards in SWAHN I and SWAHN II

	**SWAHN I**		**SWAHN II**		
**Standard**	***n***	**Median (days)**	***n***	**Median (days)**	**Target standard (days)**
GP/GDP letter to first outpatient appointment – laryngeal cancers	143	21	160	21	14
GP/GDP letter to first outpatient appointment – oral cancers	146	11	201	11	14
					
First outpatient appointment to joint head and neck clinic – laryngeal cancers	77	28	122	30	14
First outpatient appointment to joint head and neck clinic – oral cancers	87	14	167	18	14
					
First outpatient appointment to surgery – laryngeal cancers	37	26	29	28	
First outpatient appointment to surgery – oral cancers	103	29	140	38	
					
First outpatient appointment to radiotherapy – laryngeal cancers	84	56	125	64	
First outpatient appointment to radiotherapy – oral cancers	34	42	56	48	

GP=general practitioner; GDP=general dental practitioner.

). While the time between general practitioner (GP) letter and first outpatient visit remained short for oral cancer (10 days both audits), it remained long for laryngeal cancer (21 days both audits; target 14 days). However, the range diminished for both between audits. Median times between first hospital visit and definitive surgery increased from 26 to 28 days for laryngeal cancer and 29 to 38 days for oral cancer. For radiotherapy, corresponding waits were 56 and 64 days (larynx) and 42 and 47.5 days (oral cavity).

### 2-year survival

Comparison between 2-year survival figures for SWAHN I and SWAHN II is shown in Table 4

**Table 4:** Kaplan–Meier crude survival at 2 years – comparison between SWAHN I and SWAHN II, including comparison between individual cancer networks

	**2-year survival – SWAHN I**		**2-year survival – SWAHN II**	
	**Survival**	**Confidence intervals**	**Survival**	**Confidence intervals**
	**%**	**%**	**%**	**%**
Overall	64.1	60.2–68.0	65.1	61.6–68.6
				
Age group (years)				
0–64	74.8	69.1–80.5	70.6	65.7–75.5
65–74	62.5	55.2–69.8	65.3	58.2–72.4
75+	51.2	43.6–58.8	57.0	50.5–63.5
				
Sex				
Male	66.6	61.7–71.5	65.5	61.2–69.8
Female	59.9	53.2–66.6	64.2	58.1–70.3
				
Stage				
I	89.8	84.3–95.3	87.0	81.5–92.5
II	68.2	58.2–78.2	81.0	74.1–87.9
III	55.3	45.3–65.3	60.2	51.2–69.2
IV	46.8	39.4–54.2	45.6	39.3–51.9
Unknown	68.8	59.6–78.0	67.7	58.1–77.3
				
Site				
Larynx	72.1	65.4–78.8	84.7	79.6–89.8
Oral	62.4	55.1–69.7	62.2	56.1–68.3
Pharynx	51.3	42.3–60.3	50.1	42.3–57.9
Salivary gland	72.0	59.5–84.5	69.6	57.6–81.6
Other	64.6	51.1–78.1	51.9	40.9–62.9
				
Network				
Three counties	63.4	52.2–74.6	67.6	58.8–76.4
ASWCS	59.7	50.9–68.5	62.9	56.2–69.6
Central south coast	66.9	59.3–74.5	68.7	61.6–75.8
Dorset	55.9	43.2–68.6	61.8	48.9–74.7
Peninsula	68.1	61.0–75.2	63.7	56.8–70.6
				
Waiting times – GP to outpatient (weeks)				
<2	59.0	52.5–65.5	61.0	55.1–66.9
>2	70.7	64.8–76.6	71.9	66.8–77.0

ASWCS=Avon, Somerset and Wiltshire Cancer Services.

. Overall crude survival was 64.1% in 1997 and 65.1% in 2000. In both cohorts, survival was significantly affected by age, stage and site. For both SWAHN I and II, 2-year survival was significantly better if patients took longer between GP letter and first outpatient visit than the government's target time of 2 weeks (or 10 working days). A Cox proportional hazard analysis of SWAHN I 2-year survival data showed a marked trend towards improved survival if the patient had been assessed in a multidisciplinary clinic (*P*=0.1). However, survival was significantly higher for those who had had a pretreatment chest X-ray (hazard ratio 0.7, *P*=0.03). A similar analysis of SWAHN II 2-year survival data showed significantly improved survival for patients assessed in a multidisciplinary clinic (hazard ratio 0.7, *P*=0.02) and those receiving a pretreatment chest X-ray (hazard ratio 0.7, *P*=0.03).

There was no significant difference in 2-year survival in either cohort with respect to age, sex or cancer network. A significant improvement in survival was indicated for stage II disease (68.2% in 1997, 81% in 2000, log-rank test 4.5, *P*=0.03) and for laryngeal carcinoma (72.1% in 1997, 84.7 in 2000, log-rank test 8.41, *P*<0.01). However, this latter improvement did not persist when multiregression analysis was conducted to take account of case-mix.

### Quality of life

No patients in SWAHN I had quality of life measurements recorded. In the second cohort, 32 of 727 cases had such measurements, all using the EORTC head and neck tool.

## DISCUSSION

### Principal findings

In the south and west of England in the late 1990s, there was an improvement in standards for the process of head and neck cancer care as measured against consensus standards. The biggest improvement was in the number of patients being assessed in a multidisciplinary clinic. However, ‘waiting’ times increased. There was increasing experience among radiotherapists, but for surgeons the median number of cases treated annually remained 4. Only one network showed evidence of ‘centralisation’ of treatment. Of the five ‘key quality indicators’ studied, assessment at a multidisciplinary clinic and performance of a pretreatment chest X-ray were associated with significantly improved survival. There was no significant difference in outcomes between cancer networks.

### Weaknesses

There was probably incomplete coverage, as numbers fell short of actual death registrations. Both were paper-based exercises, and not all questionnaires were completed by consultants, which may have introduced errors despite attempts at quality control. Performance status has major effects on survival but was rarely reported and the external validity of the chosen system is now doubtful (Aronson *et al*, 2003). Likewise, the low rates of recording of quality of life (0 and 4% respectively) made assessment of the effect of performance improvements on this important measure impossible. It was not the intent of this study to investigate treatment morbidity, and this should be addressed by future audits. Assessing survival at 2 years may be seen as premature. However, a 5-year analysis for patients in SWAHN I (data not shown) shows that the trends at 2 years exactly reflect those at 5 years. Skin, lip and thyroid cancer cases are important parts of head and neck oncology workload, but the consensus view was that the management of these cases was distinct from that of ‘core’ head and neck cancers.

The cost of collecting data was £40.00 per case in 1997 and £50.00 per case in 2000. The provision of a national, centralised electronic data collection system, presently being piloted, may reduce these *per capita* costs significantly in the future.

### Standards

It was gratifying that there had been a rapid increase in all nontemporal standards between 1997 and 2000, especially access to multidisciplinary clinics. However, in all consensus documents on cancer (Expert Advisory Group on Cancer, 1995; Wilson, 2003), such access is regarded as a fundamental right, so there is still considerable scope for improvement. Similarly, it is difficult to see how an accurate treatment plan may be drawn up in the absence of TNM staging (more than 10%). The use of chemotherapy outside clinical trials is common (one in 11 patients) with little standardisation of protocols.

### Process times

Increases in process time were also worrying. One explanation is the increase in resource use, particularly radiology, pathology and theatre access, required to comply with care standards. This would be exacerbated by ‘creeping centralisation’ into fewer centres, which are not adequately funded to support this change. However, only two networks showed signs of having centralised in this way, and, probably as a result, the number of patients treated per clinician remained as tiny as in a 1997 study (Edwards *et al*, 1997). The use of care/critical pathway techniques (Wheelwright *et al*, 2002) might help identify where the rate-limiting steps are occurring in each Trust. Alternatively, the provision of more facilities by the recent cash injection into cancer services may have shortened times post-2000 (Department of Health, 2002). The next audit cycle should shed light on this.

### 2-year survival rates

The 2-year survival rates for the south and west of England are comparable to those published for the USA (Shirinian *et al*, 1994; Rosenthal *et al*, 2002) and the UK (Robin *et al*, 1991; Woolgar *et al*, 1999). Since the ‘quality’ of care in the region appeared to have improved between 1997 and 2000, it was disappointing that this was not reflected in improved survival figures overall. It is possible that gains achieved by better radiology, endoscopy, staging and multidisciplinary planning were offset by lengthened waiting times for investigation and treatment: ‘delays’ in treatment have been previously shown to affect survival adversely (Rosenthal *et al*, 2002).

It is presently mandatory for all hospital Trusts to produce regular data on the proportions of suspected cancer patients being seen by a specialist within 2 weeks of GP referral as a major performance target. However, we found that, in practice, being seen within the government's 2-week target time resulted in a significantly poorer survival rate at 2 years. Further analysis of these data showed that this was explained by a trend towards higher tumour stage in the 2-week group compared to those waiting longer. This is a reasonable explanation as it is easier for general practitioners to ‘spot’ bigger tumours than small ones with often vague symptoms. However, these data cast a slight shadow over the Government's reliance on this temporal target.

A trend in SWAHN I towards improved survival if patients had been assessed before treatment in a multidisciplinary clinic became significant in SWAHN II. The need for multidisciplinary assessment in cancer care has been a clarion call, despite difficulties in designing trials to prove its worth (Hanks *et al*, 2002). This is the first time a significant impact of such clinics on survival has been demonstrated for head and neck oncology.

We have also twice demonstrated an unexpected association between the performance of a pretreatment chest X-ray and improved survival. Possible explanations for this include missing significant chest disease, the use of CT scans in advanced disease or X-ray use being a surrogate marker of ‘quality’ of care.

### Meaning of the study

We have measured improvement in the quality of the care process for patients with head and neck cancer in southwest England. However, lengthened process time may have prevented this being translated into improved survival. Policy makers must plan for the knock-on effects of adherence to quality standards and increasing centralisation of care. At present, both are happening without the necessary plans and funds to accommodate them. The survival advantage gained by persons assessed in a multidisciplinary manner strongly supports the end of single clinician practice.

### Unanswered questions and future research

Future studies will examine whether these trends for standards and centralisation continue and are translated to increased 2- and 5-year survival. Other studies should address the reasons for the increasing incidence of this cancer and perform more detailed examinations of the effect service configuration has on patient outcomes.
